# CpG‐related SNPs in the MS4A region have a dose‐dependent effect on risk of late–onset Alzheimer disease

**DOI:** 10.1111/acel.12964

**Published:** 2019-05-29

**Authors:** Yiyi Ma, Gyungah R. Jun, Jaeyoon Chung, Xiaoling Zhang, Brian W. Kunkle, Adam C. Naj, Charles C. White, David A Bennett, Philip L. De Jager, Alzheimer’s Disease Genetics Consortium, Richard Mayeux, Jonathan L. Haines, Margaret A. Pericak‐Vance, Gerard D. Schellenberg, Lindsay A. Farrer, Kathryn L. Lunetta

**Affiliations:** ^1^ Department of Medicine (Biomedical Genetics) Boston University School of Medicine Boston Massachusetts; ^2^ Center for Translational & Computational Neuroimmunology, Multiple Sclerosis Clinical Care and Research Center, Division of Neuroimmunology, Department of Neurology Columbia University Medical Center New York New York; ^3^ Department of Biostatistics Boston University School of Public Health Boston Massachusetts; ^4^ Department of Ophthalmology Boston University School of Medicine Boston Massachusetts; ^5^ John P. Hussman Institute for Human Genomics, Miller School of Medicine University of Miami Miami Florida; ^6^ Department of Biostatistics, Epidemiology, and Informatics University of Pennsylvania Perelman School of Medicine Philadelphia Pennsylvania; ^7^ Department of Pathology and Laboratory Medicine University of Pennsylvania Philadelphia Pennsylvania; ^8^ Program in Translational NeuroPsychiatric Genomics Institute for the Neurosciences, Departments of Neurology and Psychiatry, Brigham and Women's Boston Massachusetts; ^9^ Program in Medical and Population Genetics Broad Institute Cambridge Massachusetts; ^10^ Rush Alzheimer’s Disease Center Rush University Medical Center Chicago Illinois; ^11^ Department of Neurology and Sergievsky Center Columbia University New York New York; ^12^ Department of Epidemiology and Biostatistics Case Western Reserve University Cleveland Ohio; ^13^ Department of Neurology Boston University School of Medicine Boston Massachusetts; ^14^ Department of Epidemiology Boston University School of Public Health Boston Massachusetts

**Keywords:** Alzheimer disease, DNA methylation, epigenetics, eQTL, genetics, mQTL

## Abstract

CpG‐related single nucleotide polymorphisms (CGS) have the potential to perturb DNA methylation; however, their effects on Alzheimer disease (AD) risk have not been evaluated systematically. We conducted a genome‐wide association study using a sliding‐window approach to measure the combined effects of CGSes on AD risk in a discovery sample of 24 European ancestry cohorts (12,181 cases, 12,601 controls) from the Alzheimer's Disease Genetics Consortium (ADGC) and replication sample of seven European ancestry cohorts (7,554 cases, 27,382 controls) from the International Genomics of Alzheimer's Project (IGAP). The potential functional relevance of significant associations was evaluated by analysis of methylation and expression levels in brain tissue of the Religious Orders Study and the Rush Memory and Aging Project (ROSMAP), and in whole blood of Framingham Heart Study participants (FHS). Genome‐wide significant (*p* < 5 × 10^−8^) associations were identified with 171 1.0 kb‐length windows spanning 932 kb in the *APOE* region (top *p* < 2.2 × 10^−308^), five windows at *BIN1* (top *p* = 1.3 × 10^−13^), two windows at *MS4A6A* (top *p* = 2.7 × 10^−10^), two windows near *MS4A4A* (top *p* = 6.4 × 10^−10^), and one window at *PICALM* (*p* = 6.3 × 10^‐9^). The total number of CGS‐derived CpG dinucleotides in the window near *MS4A4A* was associated with AD risk (*p* = 2.67 × 10^−10^), brain DNA methylation (*p* = 2.15 × 10^−10^), and gene expression in brain (*p* = 0.03) and blood (*p* = 2.53 × 10^−4^). Pathway analysis of the genes responsive to changes in the methylation quantitative trait locus signal at *MS4A4A* (cg14750746) showed an enrichment of methyltransferase functions. We confirm the importance of CGS in AD and the potential for creating a functional CpG dosage‐derived genetic score to predict AD risk.

## 1 INTRODUCTION

Much has been learned about the genetic basis of Alzheimer disease (AD), the most common cause of dementia in the elderly. Genome‐wide association studies (GWAS) have identified common and rare variants in more than 30 loci that contribute to AD risk (Bellenguez et al., 2017; Hollingworth et al., 2011; Jakobsdottir et al., 2016; Jun et al., 2017, 2016; Lambert et al., 2013; Mez et al., 2017; Naj et al., 2011; Sims et al., 2017). However, these associations explain only a fraction of the heritability of AD, and their functional consequence also remains unclear (Lambert et al., 2013; Ridge, Mukherjee, Crane, & Kauwe, 2013). Thus, here we investigate AD risk from a different perspective.

Epigenetic phenomena such as DNA methylation may be involved but have not been studied extensively in AD. DNA methylation is intimately associated with genetic variation because of frequent attachment of a methyl group directly to a DNA nucleotide, particularly a dinucleotide comprising a cytosine and guanine (CpG). CpG‐related SNPs (CGS) alter the sequence of the primary target sites for DNA methylation (Lister et al., 2009) and account for a significant fraction (~38%–88%) of allele‐specific methylation (ASM) regions in the human genome (Shoemaker, Deng, Wang, & Zhang, 2010). It has been demonstrated that more than 80% of CGSes have a regulatory role in DNA methylation (Zhi et al., 2013). Recently, we found that a haplotype of multiple CGSes is associated with DNA methylation patterns on a genome‐wide scale (Ma et al., 2016). DNA methylation has been shown to influence risk of age‐related diseases (Hunter et al., 2012; De Jager et al., 2014). For example, a genome‐wide DNA methylation study reported association of AD pathological features with methylation changes at several loci (De Jager et al., 2014). Also, levels of DNA methylation of *GSTM1* and *GSTM5* have been associated with risk of age‐related macular degeneration (Hunter et al., 2012).

In this study, we evaluated the association of AD with CGSes genome‐wide and validated significant findings by expression quantitative trait locus (eQTL) and methylation QTL (mQTL) analyses.

## 2 RESULTS

### 2.1 Sliding Window Association of CGSes with AD

Association of AD with CGSes was tested genome‐wide using sliding windows that were 1 kb in length, overlapping by 0.5 kb and contained at least two CGSes. These analyses, which were performed using SKAT‐O (Lee, Wu, & Lin, 2012), considered the combined effects of all CGSes in a window and weighted rare variants more heavily than common variants. Because the SKAT‐O window‐based test does not consider the effect direction of the variants in each window, we also tested a model including CGS dosage which was calculated as the total number of CpG dinucleotides created by the CGSes in the window. Genome‐wide analysis of 2,288,371 overlapping windows each containing at least two CGSes showed little evidence of inflation (λ = 1.099, Figure S1). SKAT‐O and CGS dosage approaches provided similar results across the genome (Figure S2) including five distinct genome‐wide significant loci with windows at *BIN1* (SKAT‐O *p* = 1.27 × 10^−13^, CGS dosage *p* = 4.74 × 10^−18^), *MS4A6A* (SKAT‐O *p* = 2.66 × 10^−10^, CGS dosage *p* = 3.40 × 10^−10^), *MS4A4A* (SKAT‐O *p* = 6.36 × 10^−10^, CGS dosage *p* = 2.67 × 10^−10^), *PICALM* (SKAT‐O *p* = 6.34 × 10^−9^, CGS dosage *p* = 1.42 × 10^−9^), and *APOE* (SKAT‐O *p* = 2.99 × 10^−46^, CGS dosage *p* = 2.77 × 10^−556^) (Table 1). Although the top windows at *BIN1* and *PICALM* identified by SKAT‐O do not reach genome‐wide significance in the CGS dosage test, the CGS dosage test identified significant associations with other windows at these loci. Windows at *LRFN2‐UNC5CL* and *TREM2* are genome‐wide significant with only the SKAT‐O test, whereas the windows at *CR1* are genome‐wide significant with only the CGS dosage test. All genome‐wide significant windows identified by SKAT‐O were replicated (Table 2).

**Table 1:** Top‐ranked windows associated with AD by SKAT‐O and CG dosage methodologies in discovery stage

Chr	Gene	Start	End	*N* of CGSes	P range of CGSes (min, max)	Window P^a^	Window P^b^	Beta (*SE*)
Common loci identified by two methods								
2	BIN1	127,847,001	127,848,000	2	(1.48E−13, 5.88E−06)	1.27E−13	2.14E−03	−0.02 (0.005)
2	BIN1	127,881,001	127,882,000	2	(2.5E−12, 3.67E−03)	1.09E−03	4.74E−18	0.18 (0.02)
11	MS4A6A	59,923,001	59,924,000	2	(1.41E−10, 1.25E−09)	2.66E−10	3.40E−10	−0.01 (0.002)
11	MS4A4A	60,087,501	60,088,500	2	(8.44E−12, 1.23E−05)	6.36E−10	2.67E−10	−0.02 (0.003)
11	PICALM	85,759,501	85,760,500	2	(3.77E−05, 0.11)	6.34E−09	9.28E−05	0.01 (0.002)
11	PICALM	85,845,001	85,846,000	2	(3.77E−05, 0.11)	5.10E−02	1.42E−09	0.13 (0.02)
19	APOE	45,411,501	45,412,500	2	(<2.23e−308, 3.56E−28)	2.99E−46	2.77E−556	0.2 (0.004)
Top loci identified by either method								
1	CR1	207,737,501	207,738,500	3	(1.49E−10, 0.06)	7.01E−04	8.57E−11	−0.15 (0.02)
6	LRFN2‐UNC5CL	40,825,501	40,826,500	3	(1.38E−06, 0.90)	1.21E−08	8.00E−02	−0.01 (0.005)
6	TREM2	41,128,501	41,129,500	5	(1.34E−06, 0.92)	1.73E−08	6.80E−06	−0.08 (0.002)

a P values obtained by SKAT‐O test.

b P values obtained by CGSes dosage test and beta represent the change in log odds of AD per 1‐unit increase in dosage of CpG dinucleotides comprising the CpG‐related SNPs in the window.

**Table 2:** Top‐ranked windows associated with AD in replication stage

Window	Chr	Start	End	Region or closest gene	*N* of CGSes	Top CGS in the window			Discovery stage			Replication stage		
						rsID	Effect allele	MAF	P for window	OR (95% CI) for top CGS	P for top CGS	P for window	OR (95% CI) for top CGS	P for top CGS
1	2	127,847,001	127,848,000	*BIN1*	2	rs35114168	A	0.39	1.27E−13	1.16 (1.12, 1.21)	1.48E−13	2.96E−07	1.13 (1.08, 1.18)	2.96E−07
2	11	59,923,001	59,924,000	*MS4A6A*	2	rs983392	G	0.39	2.66E−10	0.88 (0.85, 0.91)	1.41E−10	2.83E−05	0.91 (0.87, 0.95)	2.53E−05
3	11	60,087,501	60,088,500	*MS4A4A/MS4A6E*	2	rs4354705	C	0.36	6.36E−10	0.87 (0.83, 0.90)	8.44E−12	6.93E−03	0.94 (0.90, 0.98)	3.97E−03
4	11	85,759,501	85,760,500	*PICALM*	2	rs694011	T	0.32	6.34E−09	0.90 (0.86, 0.95)	3.77E−05	1.65E−05	0.90 (0.86, 0.94)	9.30E−06
5	19	45,411,501	45,412,500	*APOE*	2	rs429358	C	0.25	2.99E−46	3.73 (3.53, 3.94)	<2.23E−308	<2.23E−308	3.47 (3.26, 3.69)	1.09E−345

**Figure 1:**
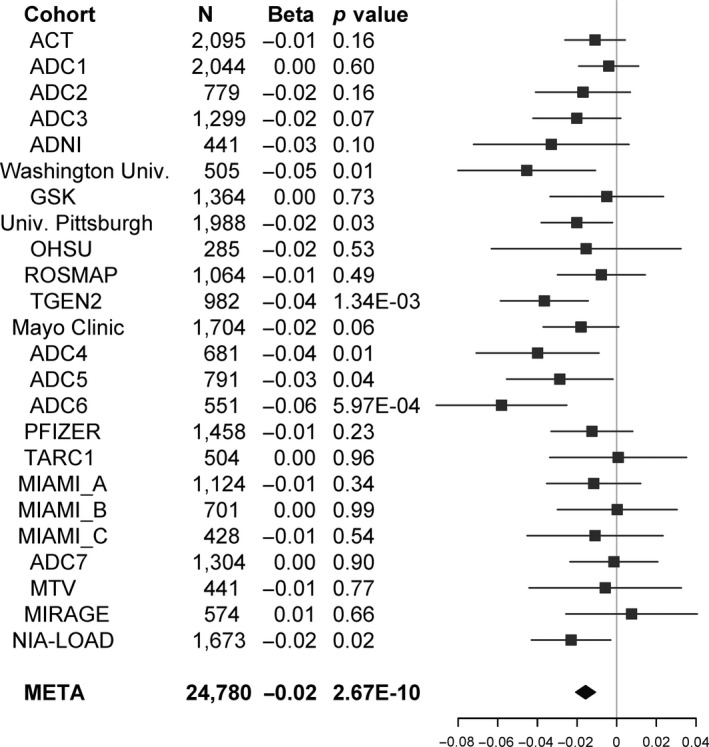
Forest plot of dose–response effect of the number of CpG dinucleotides created by the CGSes in the intergenic window close to *MS4A4A* on the logged odds ratio of AD. The filled square and horizontal line for each population or the filled diamond for the summary denote the estimated logged odds ratio and its 95% CI per unit increase in the number of CpG dinucleotides in the window

Windows in *MS4A4A* and *MS4A6A* showed a strong negative dosage effect of CpG dinucleotides on AD risk (change in log odds of AD = −0.01 and −0.02 per one unit dinucleotide increase, *p* = 2.67 × 10^−10^ and 3.4 × 10^−10^, respectively). This effect was evident in 18 out of 24 cohorts (Figure 1). The dosage of CpG dinucleotides created by the two CGSes in the *APOE* window has significantly positive association with AD risk (change in log odds of AD = 0.2 per one unit dinucleotide increase, *p* = 2.77 × 10^−556^).

In order to show the unique role of CGSes in these windows, we compared the significance level for the windows under two conditions, (a) including only CGSes and (b) including only non‐CGSes, which are the SNPs that do not disrupt CpG dinucleotide formation. As shown in Table 3, the *p* values of all the identified AD‐associated windows in Table 1 were attenuated when only non‐CGSes were included in the test. The number of CGSes and non‐CGSes in each top window differed by no more than two except for the windows at *MS4A4A* and *PICALM*. There is very modest LD between the top CGS and non‐CGS for the most of the windows except for a window at *MS4A6A* (*R* ^2^ = 0.98) which is 165 kb from the top window at *MS4A4A* (*R* ^2 ^= 0.37). The attenuation of the significance level was also observed at the individual SNP level for the comparisons of the two types of SNPs in each window, noting that the *APOE* region did not contain any non‐CGSes (Table S4).

**Table 3:** Comparisons of the top windows containing CGSes versus non‐CGSes

Chr	Start	End	Gene	P of window		*N* of variants		LD between CGS and non‐CGS (R^2^)	
				CGS	Non‐CGS	CGS	Non‐CGS	Top CGS and non‐CGS	Any pairs (min, max)
2	127,847,001	127,848,000	BIN1	1.27E−13	3.30E−05	2	2	0.01	(2.38E−05, 0.01)
11	59,923,001	59,924,000	MS4A6A	2.66E−10	0.786	2	4	0.98	(8.73E−03, 0.98)
11	60,087,501	60,088,500	MS4A4A	6.36E−10	0.029	2	9	0.37	(5.99E−04, 1)
11	85,759,501	85,760,500	PICALM	6.34E−09	0.035	2	6	0.15	(1.33E−03, 0.24)
19	45,411,501	45,412,500	APOE	2.99E−46	NA	2	0	NA	NA

### 2.2 Association of CGSes with DNA methylation and gene expression

Windows containing CGSes located in *MA4A4A*, *PICALM* and *APOE* were associated (*p* ≤ 0.05) with the degree of DNA methylation in brains (Table 4); however, only the *MS4A4A* window was significantly associated in brains after correction for the 176 methylation probes tested for association (adjusted *p* = 2.15 × 10^−9^ at cg14750746). This window was also nominally associated with increased methylation in blood after correcting for the same 176 methylation probes (nominal *p* = 3.34 × 10^−4^ and adjusted *p* = 0.06). In addition, the number of CpG dinucleotides created by the CGSes in the intergenic window between *MS4A4A* and *MS4A6E* was associated with increased expression of *MS4A4A* in both brain (*p* = 0.03) and blood (*p* = 2.53 × 10^−4^). The *MS4A6A* window was associated with DNA methylation (adjusted *p* = 1.47 × 10^−7^) and gene expression (*p* = 5.89 × 10^−26^) in blood, but rs12226022 was not well imputed in the ROSMAP dataset to test this association in brain.

**Table 4:** Association between CGSes and methylation and gene expression

Gene	Position	Name	Methylation of CpG site							Gene expression				
			Brain				Blood			Brain			Blood	
			Beta (*SE*)^a^	P1^a^	P2^a^	*P* b	Beta (*SE*)^a^	P1^a^	P2^a^	Beta (*SE*)^a^	*p* a	*P* b	Beta (*SE*)^a^	*p* a
BIN1	127,800,646	cg00436254	−1.87E−04 (2.28E−03)	0.93	1.00	2.08E−03	−5.48E−03 (6.40E−04)	1.97E−17	5.56E−15	2.40 (2.27)	0.29	0.41	−8.40E−03 (4.15E−03)	0.04
MS4A6A	59,824,541	cg01917716	NA	NA	NA	NA	−2.91E−03 (4.72E−04)	8.13E−10	1.47E−07	NA	NA	NA	−0.04 (3.55E−03)	5.89E−26
MS4A4A	60,101,475	cg14750746	5.60E−03 (8.11E−04)	1.22E−11	2.15E−09	0.03	−2.49E−03 (6.93E−04)	3.34E−04	0.06	0.14 (0.07)	0.03	0.07	0.09 (0.02)	2.53E−04
PICALM	85,566,560	cg15822411	−3.42E−03 (1.77E−03)	0.05	1.00	1.00	−6.97E−04 (4.10E−04)	0.09	1.00	3.14E−03 (0.41)	0.99	0.76	7.68E−03 (5.11E−03)	0.13
APOE	45,395,297	cg02613937	−9.96E−04 (4.19E−04)	0.02	1.00	0.42	−4.25E−03 (2.90E−03)	0.14	1.00	−2.93 (6.19)	0.64	0.60	6.94E−03 (6.13E−03)	0.26

a Statistics obtained from CGSes dosage tests. *P*1 represents uncorrected *p*‐values, and *P*2 represents Bonferroni corrected *p*‐values calculated by multiplying the number of methylation probes included in the test which are within 1Mb distance to the window.

b Statistics obtained from SKAT‐O tests.

### 2.3 Pathway analysis at the *MS4A4A* window

Transcriptome analysis using RNAseq data from the Religious Order Study and Rush Memory and Aging Project (ROSMAP) brain samples was performed to identify the set of genes whose expression is influenced by methylation of CpG site cg14750746 that was associated with the dosage of *MS4A4A* CGSes (Table 4). In total, 15,508 protein‐coding genes remained in the analysis after removing genes expressed in less than 10% subjects. Although no genes remained significant after correcting for the number tests (threshold *p* = 3.2 × 10^−6^), there were 34 nominally associated genes (*p* < 5×10^‐3^) (Table S5) and pathway analysis showed enrichment in methyltransferase activity (Table 5).

**Table 5:** Enrichment of methyltransferase activities in the regulatory network of MS4A cluster‐associated CpG site (cg14750746) in brain using Gene Ontology (GO) terms

GO term ID	GO term description	P	FDR
GO:0050313	sulfur dioxygenase activity	8.31E−04	0.03
GO:0008276	protein methyltransferase activity	1.19E−03	0.03
GO:0008170	N‐methyltransferase activity	1.23E−03	0.03
GO:0070905	serine binding	1.66E−03	0.03
GO:0003713	transcription coactivator activity	1.69E−03	0.03
GO:0004843	ubiquitin‐specific protease activity	2.44E−03	0.03
GO:0042799	histone methyltransferase activity (H4‐K20 specific)	2.49E−03	0.03
GO:0019783	ubiquitin‐like protein‐specific protease activity	2.95E−03	0.03
GO:0036459	ubiquitinyl hydrolase activity	3.01E−03	0.03
GO:0008234	cysteine‐type peptidase activity	8.70E−03	0.06
GO:0008139	nuclear localization sequence binding	9.11E−03	0.06
GO:0008168	methyltransferase activity	9.91E−03	0.06
GO:0016741	transferase activity, transferring one‐carbon groups	0.01	0.06
GO:0003756	protein disulfide isomerase activity	0.02	0.08
GO:0016864	intramolecular oxidoreductase activity, transposing S‐S bonds	0.02	0.08
GO:0005096	GTPase activator activity	0.02	0.08
GO:0016702	oxidoreductase activity, acting on single donors with incorporation of molecular oxygen, incorporation of two atoms of oxygen	0.02	0.08
GO:0016701	oxidoreductase activity, acting on single donors with incorporation of molecular oxygen	0.02	0.08
GO:0030695	GTPase regulator activity	0.02	0.08
GO:0005048	signal sequence binding	0.02	0.08
GO:0060589	nucleoside‐triphosphatase regulator activity	0.02	0.09
GO:0018024	histone‐lysine N‐methyltransferase activity	0.03	0.09
GO:0016278	lysine N‐methyltransferase activity	0.03	0.10
GO:0016279	protein‐lysine N‐methyltransferase activity	0.03	0.10

## 3 DISCUSSION

Our study using a sliding‐window approach confirmed the importance of CGS in AD and is the first to report dosage effects of CpG dinucleotides created by CGSes on AD risk. In particular, we identified six windows with a significant effect of the number of CpG dinucleotides on AD risk, including a novel and robust dose‐dependent effect in an intergenic window located between *MS4A4A* and *MS4A6E*. The number of CpG dinucleotides created by the CGSes within this window is inversely associated with the risk of AD. The potential functional importance of CGSes in AD is supported by evidence showing that the significance of almost all of the top windows was attenuated when non‐CGSes were included instead of CGSes. This observation does not seem to be related to the differences in the number of variants or LD between CGSes and non‐CGSes.

The *MS4A* gene cluster encodes a family of proteins spanning the cellular membrane four times which share similar polypeptide sequence and predicted topological structure. *MS4A6A* expression in brain is positively associated with AD‐related neurofibrillary tangles and neuritic plaques (Karch et al., 2012; Martiskainen et al., 2015). AD risk alleles at these loci were reported to be associated with higher expression in brain (Allen et al., 2012; Karch et al., 2012; Martiskainen et al., 2015). The underlying mechanism for the effects of *MS4A* genes on AD may be related to their regulation of calcium channels (Walshe et al., 2008), immune system (Zuccolo et al., 2013). Our findings of an association of the CpG dinucleotide dosage in this region with AD risk suggest a potential novel AD‐related mechanism involving *MS4A* genes. Further experiments examining DNA methylation in the *MS4A* region are necessary to clarify the exact mechanism.

All of the loci identified in our study using a sliding‐window approach were previously reported to be associated with AD through DNA methylation analyses, indicating an overlap between genetic and epigenetic mechanisms. For example, brain DNA methylation levels of CpG sites located in the top‐ranked loci have been associated with clinical and pathological diagnoses of AD in a sample of 740 ROSMAP participants, many of whom are included in the ADGC GWAS dataset (De Jager et al., 2014). The mQTL CpG sites identified in our study are correlated with the previously reported (De Jager et al., 2014) AD‐associated CpG sites in both brain and blood (all *p* < 0.05) (Table S6), but it is unclear why the pairs of methylation probes in *MS4A* region and *APOE* are inversely correlated in brain and blood.

All of the genes identified by our analyses have been implicated in inflammation and the immune system. *BIN1* knock‐out mice were shown to have higher incidence of inflammation during aging (Chang et al., 2007). *BIN1* was also reported to be related to inflammation and immunity by its participation in the phagocytic pathway (Gold et al., 2004) and regulation of critical enzymes against pathogens (Muller, DuHadaway, Donover, Sutanto‐Ward, & Prendergast, 2005). Genes in the *MS4A* family have been shown to activate T cells and trigger production of inflammatory cytokines (Yan et al., 2013). Expression of *PICALM* was reduced in subjects who underwent gastric bypass surgery to reverse their pro‐inflammatory state of obesity (Ghanim et al., 2012), and *PICALM* overexpression in vitro was found to reduce the endosomal localization of the mannose‐6‐phosphate receptor (M6PR) which binds to the herpes virus (Brunetti, Dingwell, Wale, Graham, & Johnson, 1998). It is still controversial whether *APOE‐*ε4 causes anti‐ or pro‐inflammatory effects, but it is generally accepted that *APOE* is related to inflammation (Dorey, Chang, Liu, Yang, & Zhang, 2014). Our collective findings suggest that DNA methylation may be a molecular mechanism underlying aberrant inflammatory responses related to AD.

Our findings also suggest that the sliding‐window approach focused on CGSes is useful for identifying loci whose influence on disease risk may involve clinically relevant epigenetic mechanisms. In the large GWAS conducted by the International Genomics of Alzheimer's Project (Lambert et al., 2013), approximately 44% of the top AD‐associated SNPs are CGSes. However, not all of these CGSes were significantly associated with AD in our analysis (e.g., CGSes in *CR1, CD2AP*, and *CLU*; Table S1). Interestingly, none of these three loci were reported to have significant brain methylation changes related to AD pathology (De Jager et al., 2014), indicating that their effects on AD may not involve DNA methylation.

Our study has several limitations. All the identified top windows for AD were previously reported loci associated with AD (Guerreiro et al., 2013; Jonsson et al., 2013; Lambert et al., 2013; Naj et al., 2011). This was expected because the samples of our and previously published GWAS are highly overlapping. However, our study ascribes potential function to some of these results, especially those occurring in noncoding regions. In order to identify the relative importance of the CGSes in the top windows compared to non‐CGSes, we performed conditional analysis adjusting for the top GWAS SNP. For all windows, the association signal for both the GWAS SNP and CpG dosage was attenuated when both were included in the model. In particular, for the intergenic window between *MS4A4A* and *MS4A6E*, the *p*‐values for both CpG dosage and the GWAS variant had similar reduction in significance (Table S7). The squared correlation (*r* ^2^) between the GWAS variant and the CGS with the largest influence on the dosage effect in *MS4A4A* window is 0.56. Thus, it is not possible to conclude from the conditional analysis whether the GWAS variant, the window CpG dosage, or another variant in the region that is correlated with both of these markers, is responsible for the association. We did not remove CGSes in high LD, which may inflate the number of significant findings. However, some of these associations may be independent because multiple adjacent methylated CpG sites can serve as the platform for chromatin binding proteins that lead to changes in chromatin state (Bartke et al., 2010). Another concern is that despite experimental evidence suggesting an optimal window size of 1kb, it is unknown whether other window sizes may increase power. Also, our selection of the default weights of variants has bias toward rare variants. Finally, we observed that the CGS most significantly associated with AD risk also has significant mQTL and eQTL effects that survive regional multiple test correction but do not achieve genome‐wide significance.

In conclusion, we confirmed the importance of CGS in AD and the potential for creating a functional genetic score based on CpG dosage to predict disease risk. However, it is unknown whether these CGS signals act as causative mechanisms in AD progression. Further replication and mechanistic studies are necessary to validate these findings. Future genome‐wide mQTL and eQTL analyses may extend our findings.

## 4 EXPERIMENTAL PROCEDURES

### 4.1 Genome‐wide association analysis of CGSes with AD

#### 4.1.1 CGS annotation

CGSes were annotated as described previously (Ma et al., 2016). In brief, CGS information was retrieved by Galaxy (Goecks, Nekrutenko, Taylor, & Galaxy, 2010) from UCSC human genome browser based on SNP141 and human hg19 sequence data.

#### 4.1.2 Discovery stage subjects

The discovery stage included 12,181 unrelated cases and 12,601 controls from 22 cohorts with European ancestry participating in the Alzheimer's Disease Genetic Consortium (ADGC) (Table S2). Characteristics of the ADC7 cohort are provided in the Appendix S1, and details of other study cohorts were previously described (Jun et al., 2016; Lambert et al., 2013). Studies of the individual cohorts were approved by the appropriate Institutional Review Boards, and written informed consent for all subjects was provided on behalf of themselves or for substantially cognitively impaired subjects, by a caregiver, legal guardian, or other proxy.

### 4.2 Statistical analysis

Details of SNP genotyping and quality control are described elsewhere (Jun et al., 2016; Lambert et al., 2013). SNP genotype imputation was performed using IMPUTE2 with reference haplotypes from the March 2012 release of 1,000 Genomes. Principal component (PC) analysis was conducted using the smartpca program in EIGENSOFT (Patterson, Price, & Reich, 2006; Price et al., 2006) to evaluate population substructure within each dataset. Association of AD risk with CGSes was tested using a sliding‐window approach (Tang, Feng, Sha, & Zhang, 2009). Windows spanning 1kb were constructed based on evidence suggesting that sequence variants within 1 kb can affect the methylation status of a gene (Lienert et al., 2011). Consecutive windows with a 500 bp overlap were tested to optimize power for detection of associations and ensure a sufficient number of SNPs in each window. Thus, for example, each unique 2000 bp region contains three overlapping windows. CGSes with imputation quality (*r* ^2^) ≤0.4 or genotype data available for less than half of the cohorts were removed. Windows with fewer than two CGSes were omitted from the analysis. After these filtering steps, 2,288,371 windows remained for association analyses.

The association of AD with the combined effects of multiple CGSes in each window on the risk of AD was evaluated by logistic regression using the optimal sequence kernel association test (SKAT‐O) (Lee et al., 2012) using R package seqMeta (https://cran.r-project.org/web/packages/seqMeta/index.html) as implemented in Universal Genome Analyst (UGA) software (https://github.com/rmkoesterer/uga). The fast *P* value calculation “integration” method was used as a screening tool. Windows with *p* ≤ 5 × 10^‐4^ or no reported *p* value were re‐analyzed using the “saddlepoint” method (Duchesne & de Micheaux, 2010). We used the default weights of the seqMeta package to up weight the contributions from rare variants with the aim to identify potential novel loci. The same methodology was applied to the analysis of non‐CGSes. SKAT‐O is not sensitive to effect direction of the individual variants included in the test and thus does not produce effect estimates. Thus, we also conducted the dose–response effect of the multiple CGSes in the window on AD risk using logistic regression. The allele that creates a CpG dinucleotide was considered as the effect allele and the allele that disrupts the CpG dinucleotide as the reference allele. The sum of the imputed dosages for multiple CGSes in each window was calculated and used as the exposure variable for the logistic regression model with AD status as the outcome. The summary statistics for regression coefficients and robust standard errors from each cohort were meta‐analyzed using an inverse variance‐weighted, fixed‐effects approach implemented in METAL (Willer, Li, & Abecasis, 2010). Both SKAT‐O and dosage analyses were adjusted for age, sex, and PCs. Windows surviving Bonferroni‐corrected genome‐wide significance level (*p* ≤ 5 × 10^−8^) from both methodologies were considered. The genome‐wide summary statistics from the two methodologies are provided in Table S8.

#### 4.2.1 Replication testing

Cohort‐specific GWAS summary statistics were obtained from a prior AD GWAS conducted by the IGAP consortium, which includes 7,554 unrelated cases and 27,382 controls from the Cohorts for Heart and Aging Research in Genomic Epidemiology (CHARGE) consortium, the European Alzheimer's Disease Initiative (EADI), and the Genetic and Environmental Risk in Alzheimer's Disease (GERAD) consortium (Lambert et al., 2013). The protocols and participant consent forms were approved by each institution. The combined effects of multiple CGSes in each window on AD were determined using the GATES method, implemented in the GATES R package (Li, Gui, Kwan, & Sham, 2011). This method extends the Simes test to combine the *p*‐values of the SNPs within a region into an overall regional *p* value.

### 4.3 mQTL Analysis

Brain mQTL was obtained for 740 subjects (mean age at death = 88 years, 63.6% female) from the Religious Order Study and Rush Memory and Aging Project (ROSMAP), and blood mQTL data obtained from 2,405 participants (mean age = 66 years, 54% female) of the Framingham Heart Study (FHS) Offspring cohort at examination 8 were downloaded from dbGAP (Table S3). DNA methylation profiles for both studies were measured by the Illumina Infinium HumanMethylation450 BeadChip. Analyses of FHS data were conducted in two stages. A linear mixed model was used to derive the residuals of the DNA methylation of the probe adjusted for the imputed cell types (CD8T, CD4T, NK, B‐cell, monocyte), row and column as fixed‐effects, chip ID as a random effect at first. Then, each residual was regressed on the CGSes dosage in models including age and sex as fixed‐effects and kinship matrix as random effect to account for familial correlation. Analyses of ROSMAP data were conducted with the linear model by adjusting the methylation batch, age at death, sex, post‐mortem interval, and study group (ROS or MAP), which was test to be the most appropriate model for the data as reported by De Jager et al. (2014). *p*‐values were adjusted using a Bonferroni correction for the total number of probes tested within each window.

### 4.4 eQTL analysis

Brain RNAseq data were obtained for 580 ROSMAP subjects (mean age at death = 89 years, 63.3% female), and whole blood array‐based expression data for 5,252 FHS Offspring cohort (examination 8) and Generation 3 (examination 2) participants (mean age = 55 years, 54% female) were obtained from dbGAP (Supplementary Table 3). Normalized gene expression level was regressed on the sum of dosages of CpG dinucleotides in each window with covariates for age, sex, and the first three PCs of ancestry using a linear mixed model for analyses of FHS data and a general linear model for analyses of ROSMAP data. *p*‐Values were corrected for the seven tests (i.e., 7 genes) performed.

### 4.5 Pathway analysis

Using the ROSMAP brain methylation and RNAseq data, we performed a genome‐wide expression‐methylation scan using a general linear model with the methylation of CpG site cg14750476 as the exposure variable and the normalized gene expression levels of all the protein‐coding genes as outcomes (*n* = 15,508), including the same covariates as in the mQTL and eQTL analyses. Genes with *p* < 0.005 were included in the pathway enrichment analysis implemented in the software of STRINGdb (Szklarczyk et al., 2015), which conducted a hypergeometric test, using the false discovery rate (FDR) to correct for multiple tests (Benjamini, 1995), to query the enrichment of the input gene sets against the background gene list in Gene Ontology database classified as “molecular function”.

## CONFLICT OF INTEREST

None declared.

## AUTHOR CONTRIBUTIONS

YM, KLL, and LAF wrote the manuscript. YM performed the data analysis. XZ, BWK, ACN, CCW, PLDJ, and DAB interpreted genetic association, mQTL and eQTL analyses. GRJ and JC provided technical support. RM, JLH, MAP‐V, GDS, and LAF obtained the funding for this study. KLL and LAF supervised the project. All authors read and approved the final manuscript.

## APPENDIX 1

### Members of the Alzheimer Disease Genetics Consortium

Marilyn S. Albert^1^, Roger L. Albin^2−4^, Liana G. Apostolova^5^, Steven E. Arnold^6^, Sanjay Asthana^7−9^, Craig S. Atwood^7,9^, Clinton T. Baldwin^10^, Michael M. Barmada^11^, Lisa L. Barnes^12,13^, Thomas G. Beach^14^, James T. Becker^15^, Duane Beekly^16^, David A. Benönett^12^, Eileen H. Bigio^17,18^, Thomas D. Bird^19,20^, Deborah Blacker^21,22^, Bradley F. Boeve^23^, James D. Bowen^24^, Adam Boxer^25^, James R. Burke^26^, Joseph Buxbaum^108^, Nigel J. Cairns^27^, Laura B. Cantwell^28^, Chuanhai Cao^29^, Chris S. Carlson^30^, Cynthia M. Carlsson^8^, Regina M. Carney^31^, Minerva M. Carrasquillo^32^, Steven L. Carroll^33^, Helena C. Chui^34^, Clark,^35^ Paul Crane^80^, David H. Cribbs^36^, Elizabeth A. Crocco^31^, Carlos Cruchaga^37^, Charles DeCarli^38^, F. Yesim Demirci^11^, Philip De Jager^109^, Malcolm Dick^39^, Dennis Dickson^32^, Ranjan Duara^40^, Nilufer Ertekin‐Taner^32,41^, Kelley M. Faber^42^, Kenneth B. Fallon^33^, Martin R. Farlow^43^, Steven Ferris^44^, Tatiana M. Foroud^42^, Matthew Frosch^110^, Douglas R. Galasko^45^, Marla Gearing^46,47^, Daniel H. Geschwind^48^, Bernardino Ghetti^85^, John Gilbert^70^, Jonathan D. Glass^49^, Alison M. Goate^37^, Neill R. Graff‐Radford^32,41^, Robert C. Green^50^, John H. Growdon^51^, Hakon Hakonarson^52^, Ronald L. Hamilton^53^, Kara L. Hamilton‐Nelson^70^, John Hardy^111^, Lindy E. Harrell^35^, Elizabeth Head^54^, Lawrence S. Honig^55^, Matthew J. Huentelman^112^, Christine M. Hulette^56^, Bradley Hyman^51^, Gail P. Jarvik^57,58^, Gregory A. Jicha^59^, Lee‐Way Jin^60^, Gyungah Jun^10,61,62^, M. Ilyas Kamboh^11,75^, Anna Karydas^25^, John S. K. Kauwe^63^, Jeffrey A. Kaye^64,65^, Ronald Kim,^66^ Neil W. Kowall^67,68^, Joel H. Kramer^69^, Patricia Kramer^64^, Walter Kukull^16,113^, Brian W. Kunkle^70^, Frank M. LaFerla^71^, James J. Lah^49^, Eric Larson^80^, James B. Leverenz^72^, Allan I. Levey^49^, Ge Li^73^, Andrew P. Lieberman^74^, Chiao‐Feng Lin^28^, Oscar L. Lopez^75^, Kathryn L. Lunetta^61^, Constantine G. Lyketsos^76^, Wendy J. Mack^77^, Daniel C. Marson,^35^ Eden R. Martin^17^, Frank Martiniuk^78^, Deborah Mash,^107^ Eliezer Masliah^45,79^, Wayne C. McCormick^80^, Susan M. McCurry^81^, Andrew N. McDavid^30^, Ann C. McKee^67,68^, Marsel Mesulam^18,82^, Bruce L. Miller^25^, Carol A. Miller^83^, Joshua W. Miller^60^, John C. Morris^27,84^, Shubhabrata Mukherjee^80^, Jill R. Murrell^42,85^, Amanda J. Myers^31^, Adam C. Naj^114^, John M. Olichney^38^, Vernon S. Pankratz^86^, Joseph E. Parisi^87^, Amanda Partch^28^, Henry L. Paulson^88^, William Perry^70^, Elaine Peskind^73^, Ronald C. Petersen^23^, Aimee Pierce^36^, Wayne W. Poon^39^, Huntington Potter^89^, Joseph F. Quinn^64^, Ashok Raj^29^, Murray Raskind^73^, Eric Reiman^115,116^, Barry Reisberg^44,90^, Christiane Reitz^55,91,92^, John M. Ringman^5^, Erik D. Roberson,^35^ Ekaterina Rogaeva^93^, Howard J. Rosen^25^, Roger N. Rosenberg^94^, Mark A. Sager^8^, Mary Sano^95^, Andrew J. Saykin^42,96^, Julie Schneider^12,117^, Lon S. Schneider^34,97^, William W. Seeley^25^, Amanda G. Smith^29^, Joshua A. Sonnen^98^, Salvatore Spina^85^, Peter St George Hyslop^93,99^, Robert A. Stern^67^, Rudolph E. Tanzi^51^, Tricia A. Thornton‐Wells,^100^ John Trojanowski^28^, Juan C. Troncoso^101^, Debby W. Tsuang^20,73^, Otto Valladares^28^, Vivianna M. Van Deerlin^28^, Linda J. Van Eldik^102^, Badri N. Vardarajan^55,91,92^, Harry V. Vinters^5,103^, Jean‐Paul Vonsattel^118^, Li‐San Wang^28^, Sandra Weintraub^18,104^, Kathleen A. Welsh‐Bohmer^26,105^, Jennifer Williamson^55^, Sarah Wishnek^70^, Randall L. Woltjer^106^, Clinton B. Wright^107^, Steven Younkin^32^, Chang‐En Yu^80^, and Lei Yu^12^

^1^Department of Neurology, Johns Hopkins University, Baltimore, Maryland; ^2^Department of Neurology, University of Michigan, Ann Arbor, Michigan; ^3^Geriatric Research, Education and Clinical Center (GRECC), VA Ann Arbor Healthcare System (VAAAHS), Ann Arbor, Michigan; ^4^Michigan Alzheimer Disease Center, Ann Arbor, Michigan; ^5^Department of Neurology, University of California, Los Angeles, Los Angeles, California; ^6^Department of Psychiatry, University of Pennsylvania Perelman School of Medicine, Philadelphia, Pennsylvania; ^7^Geriatric Research, Education and Clinical Center (GRECC), University of Wisconsin, Madison, Wisconsin; ^8^Department of Medicine, University of Wisconsin, Madison, Wisconsin; ^9^Wisconsin Alzheimer’s Institute, Madison, Wisconsin; ^10^Department of Medicine (Biomedical Genetics), Boston University, Boston, Massachusetts; ^11^Department of Human Genetics, University of Pittsburgh, Pittsburgh, Pennsylvania; ^12^Department of Neurological Sciences, Rush University Medical Center, Chicago, Illinois; ^13^Department of Behavioral Sciences, Rush University Medical Center, Chicago, Illinois; ^14^Civin Laboratory for Neuropathology, Banner Sun Health Research Institute, Phoenix, Arizona; ^15^Departments of Psychiatry, Neurology, and Psychology, University of Pittsburgh School of Medicine, Pittsburgh, Pennsylvania; ^16^National Alzheimer’s Coordinating Center, University of Washington, Seattle, Washington; ^17^Department of Pathology, Northwestern University Feinberg School of Medicine, Chicago, Illinois; ^18^Cognitive Neurology and Alzheimer’s Disease Center, Northwestern University Feinberg School of Medicine, Chicago, Illinois; ^19^Department of Neurology, University of Washington, Seattle, Washington; ^20^VA Puget Sound Health Care System/GRECC, Seattle, Washington; ^21^Department of Epidemiology, Harvard School of Public Health, Boston, Massachusetts; ^22^Department of Psychiatry, Massachusetts General Hospital/Harvard Medical School, Boston, Massachusetts; ^23^Department of Neurology, Mayo Clinic, Rochester, Minnesota; ^24^Swedish Medical Center, Seattle, Washington; ^25^Department of Neurology, University of California, San Francisco, San Francisco, California; ^26^Department of Medicine, Duke University, Durham, North Carolina; ^27^Department of Pathology and Immunology, Washington University, St. Louis, Missouri; ^28^Department of Pathology and Laboratory Medicine, University of Pennsylvania Perelman School of Medicine, Philadelphia, Pennsylvania; ^29^USF Health Byrd Alzheimer’s Institute, University of South Florida, Tampa, Florida; ^30^Fred Hutchinson Cancer Research Center, Seattle, Washington; ^31^Department of Psychiatry and Behavioral Sciences, Miller School of Medicine, University of Miami, Miami, Florida; ^32^Department of Neuroscience, Mayo Clinic, Jacksonville, Florida; ^33^Department of Pathology, University of Alabama at Birmingham, Birmingham, Alabama; ^34^Department of Neurology, University of Southern California, Los Angeles, Los Angeles, California; ^35^Department of Neurology, University of Alabama at Birmingham, Birmingham, Alabama; ^36^Department of Neurology, University of California, Irvine, Irvine, California; ^37^Department of Psychiatry and Hope Center Program on Protein Aggregation and Neurodegeneration, Washington University School of Medicine, St. Louis, Missouri; ^38^Department of Neurology, University of California, Davis, Sacramento, California; ^39^Institute for Memory Impairments and Neurological Disorders, University of California, Irvine, Irvine, California; ^40^Wien Center for Alzheimer’s Disease and Memory Disorders, Mount Sinai Medical Center, Miami Beach, Florida; ^41^Department of Neurology, Mayo Clinic, Jacksonville, Florida; ^42^Department of Medical and Molecular Genetics, Indiana University, Indianapolis, Indiana; ^43^Department of Neurology, Indiana University, Indianapolis, Indiana; ^44^Department of Psychiatry, New York University, New York, New York; ^45^Department of Neurosciences, University of California, San Diego, La Jolla, California; ^46^Department of Pathology and Laboratory Medicine, Emory University, Atlanta, Georgia; ^47^Emory Alzheimer’s Disease Center, Emory University, Atlanta, Georgia; ^48^Neurogenetics Program, University of California, Los Angeles, Los Angeles, California; ^49^Department of Neurology, Emory University, Atlanta, Georgia; ^50^Division of Genetics, Department of Medicine and Partners Center for Personalized Genetic Medicine, Brigham and Women’s Hospital and Harvard Medical School, Boston, Massachusetts; ^51^Department of Neurology, Massachusetts General Hospital/Harvard Medical School, Boston, Massachusetts; ^52^Center for Applied Genomics, Children’s Hospital of Philadelphia, Philadelphia, Pennsylvania; ^53^Department of Pathology (Neuropathology), University of Pittsburgh, Pittsburgh, Pennsylvania; ^54^Sanders‐Brown Center on Aging, Department of Molecular and Biomedical Pharmacology, University of Kentucky, Lexington, Kentucky; ^55^Taub Institute on Alzheimer’s Disease and the Aging Brain, Department of Neurology, Columbia University, New York, New York; ^56^Department of Pathology, Duke University, Durham, North Carolina; ^57^Department of Genome Sciences, University of Washington, Seattle, Washington; ^58^Department of Medicine (Medical Genetics), University of Washington, Seattle, Washington; ^59^Sanders‐Brown Center on Aging, Department Neurology, University of Kentucky, Lexington, Kentucky; ^60^Department of Pathology and Laboratory Medicine, University of California, Davis, Sacramento, California; ^61^Department of Biostatistics, Boston University, Boston, Massachusetts; ^62^Department of Ophthalmology, Boston University, Boston, Massachusetts; ^63^Department of Biology, Brigham Young University, Provo, Utah; ^64^Department of Neurology, Oregon Health & Science University, Portland, Oregon; ^65^Department of Neurology, Portland Veterans Affairs Medical Center, Portland, Oregon; ^66^Department of Pathology and Laboratory Medicine, University of California, Irvine, Irvine, California; ^67^Department of Neurology, Boston University, Boston, Massachusetts; ^68^Department of Pathology, Boston University, Boston, Massachusetts; ^69^Department of Neuropsychology, University of California, San Francisco, San Francisco, California; ^70^The John P. Hussman Institute for Human Genomics, University of Miami, Miami, Florida; ^71^Department of Neurobiology and Behavior, University of California, Irvine, Irvine, California; ^72^Cleveland Clinic Lou Ruvo Center for Brain Health, Cleveland Clinic, Cleveland, Ohio; ^73^Department of Psychiatry and Behavioral Sciences, University of Washington School of Medicine, Seattle, Washington; ^74^Department of Pathology, University of Michigan, Ann Arbor, Michigan; ^75^University of Pittsburgh Alzheimer’s Disease Research Center, Pittsburgh, Pennsylvania; ^76^Department of Psychiatry, Johns Hopkins University, Baltimore, Maryland; ^77^Department of Preventive Medicine, University of Southern California, Los Angeles, California; ^78^Department of Medicine–Pulmonary, New York University School of Medicine, New York, New York; ^79^Department of Pathology, University of California, San Diego, La Jolla, California; ^80^Department of Medicine, University of Washington, Seattle, Washington; ^81^Northwest Research Group on Aging, University of Washington School of Nursing, Seattle, Washington; ^82^Department of Neurology, Northwestern University Feinberg School of Medicine, Chicago, Illinois; ^83^Department of Pathology, University of Southern California, Los Angeles, Los Angeles, California; ^84^Department of Neurology, Washington University, St. Louis, Missouri; ^85^Department of Pathology and Laboratory Medicine, Indiana University, Indianapolis, Indiana; ^86^Department of Biostatistics, Mayo Clinic, Rochester, Minnesota; ^87^Department of Laboratory Medicine and Pathology, Mayo Clinic, Rochester, Minnesota; ^88^Michigan Alzheimer’s Disease Center, Department of Neurology, University of Michigan, Ann Arbor, Michigan; ^89^Department of Neurology, University of Colorado School of Medicine, Aurora, Colorado; ^90^Alzheimer’s Disease Center, New York University, New York, New York; ^91^Gertrude H. Sergievsky Center, Columbia University, New York, New York; ^92^Department of Neurology, Columbia University, New York, New York; ^93^Tanz Centre for Research in Neurodegenerative Disease, University of Toronto, Toronto, Ontario, Canada; ^94^Department of Neurology, University of Texas Southwestern, Dallas, Texas; ^95^Department of Psychiatry, Mount Sinai School of Medicine, New York, New York; ^96^Department of Radiology and Imaging Sciences, Indiana University, Indianapolis, Indiana; ^97^Department of Psychiatry, University of Southern California, Los Angeles, California; ^98^Department of Pathology, University of Washington, Seattle, Washington; ^99^Cambridge Institute for Medical Research and Department of Clinical Neurosciences, University of Cambridge, Cambridge, United Kingdom; ^100^Center for Human Genetics and Research, Department of Molecular Physiology and Biophysics, Vanderbilt University, Nashville, Tennessee; ^101^Department of Pathology, Johns Hopkins University, Baltimore, Maryland; ^102^Sanders‐Brown Center on Aging, Department of Anatomy and Neurobiology, University of Kentucky, Lexington, Kentucky; ^103^Department of Pathology & Laboratory Medicine, University of California, Los Angeles, Los Angeles, California; ^104^Department of Psychiatry, Northwestern University Feinberg School of Medicine, Chicago, Illinois; ^105^Department of Psychiatry & Behavioral Sciences, Duke University, Durham, North Carolina; ^106^Department of Pathology, Oregon Health & Science University, Portland, Oregon; ^107^Evelyn F. McKnight Brain Institute, Department of Neurology, Miller School of Medicine, University of Miami, Miami, Florida; ^108^Department of Psychiatry, Mount Sinai Hospital, New York, New York; ^109^Center for Translational & Computational Neuroimmunology, Multiple Sclerosis Clinical Care and Research Center, Division of Neuroimmunology, Department of Neurology, Columbia University Medical Center, New York, New York; ^110^C.S. Kubik Laboratory for Neuropathology, Massachusetts General Hospital, Charlestown, Massachusetts; ^111^Department of Molecular Neuroscience, University College London, London, United Kingdom; ^112^Neurogenomics Division, Translational Genomics Research Institute, Phoenix, Arizona; ^113^Department of Epidemiology, University of Washington, Seattle, Washington; ^114^Division of Epidemiology, University of Pennsylvania, Philadelphia, Pennsylvania; ^115^Arizona Alzheimer’s Consortium, Banner Alzheimer’s Institute, Phoenix, Arizona; ^116^Department of Psychiatry, University of Arizona, Phoenix, Arizona; ^117^Department of Pathology (Neuropathology), Rush University Medical Center, Chicago, Illinois; ^118^New York Brain Bank, Columbia University, New York, New York.
