# Supplementary material for: CpG‐related SNPs in the MS4A region have a dose‐dependent effect on risk of late–onset Alzheimer disease
Source: Aging Cell. 2019 May 29;18(4):e12964. doi: 10.1111/acel.12964 (PMC6612647; doi:10.1111/acel.12964)
Supplement: Supplementary file 1 [file ACEL-18-e12964-s001.docx]

**CONTENTS OF SUPPLEMENTARY MATERIAL:**

**Supplementary Text**. Description of Alzheimer Disease Center (ADC) wave 7 (ADC7) dataset included in the discovery stage

**Supplementary Table 1.** CGS annotation of reported AD-associated SNPs in Lambert et al., 2013.

**Supplementary Table 2**. Characteristic of the cohorts included in the study.

**Supplementary Table 3.** Distribution of age and sex in datasets for mQTL and eQTL analysis

**Supplementary Table 4.** Comparisons between CGS and non-CGS in each top AD-associated window in the discovery stage

**Supplementary Table 5**. Genes showing nominal association with DNA methylation level of cg14750746 in brain (P<0.005)

**Supplementary Table 6.** Pearson correlation between reported AD associated methylation site by De Jager et al., 2014 and the site reported in the current study in both brain and blood

**Supplementary Table 7**. Conditional analysis of the dosage effect of CGS on AD risk adjusted for the top GWAS SNP in the region

**Supplementary Figure 1**. Quantile-Quantile (Q-Q) plot of sliding window results in the discovery stage

**Supplementary Figure 2**. Miami plot of the P values for the associations between each window on AD risk obtained from SKAT-O (upper panel) and CG dosage tests (lower panel) in discovery stage.

**Supplementary Figure 3.** Regional plots of top AD-associated windows in discovery stage

**SUPPLEMENTAL TABLE (in tab-delimited file)**

**Supplementary Table 8**. Summary statistics of all windows on AD risk in discovery samples using both SKAT-O and CGS dosage analysis.

**Description of Alzheimer Disease Center (ADC) wave 7 (ADC7) dataset included in the discovery stage**

Subjects of ADC cohorts are ascertained and evaluated by the clinical and neuropathology cores of the 29 NIA-funded ADCs. The National Alzheimer’s Coordinating Center (NACC) coordinate the collection of samples and phenotype data collected from the 29 ADCs, implement definitions of AD cases and controls. Autopsied subjects of ADC are older than 60 years at the time of death and their detailed neuropathological data are collected. The Uniform dataset (UDS) protocol [1, 2]was used to evaluate living subjects, who were included in the study if they were documented to not have mild cognitive impairment (MCI) and were between 60 and 100 years of age at the time of the most recent assessment. AD cases met NINCDS-ADRDA criteria for definite or probable AD, or had a Clinical Dementia Rating (CDR) ≥ 1. ADC7 subjects were genotyped on the Illumina HumanOminiExpress-24 beadchip.

**Supplementary Table 1.** CGS annotation of reported AD-associated SNPs in Lambert et al., 2013[3].

| SNP | Chr | Closest gene | MAF | CGS  (yes / no) | Sequences | SNP coordinate | window coordinate (start to end) | N of CGS in window | *P* value of window with only CGSes |
| --- | --- | --- | --- | --- | --- | --- | --- | --- | --- |
| rs6656401 | 1 | CR1 | 0.20 | yes | C[A/G]T | 207692049 | NA | 1 | NA |
| rs6733839 | 2 | BIN1 | 0.41 | no | A[C/T]A | 127892810 | 127892501-127893500 | 2 | 3.44E-01 |
| rs10948363 | 6 | CD2AP | 0.27 | yes | C[A/G]A | 47487762 | 47487501-47488500 | 4 | 2.57E-01 |
| rs11771145 | 7 | EPHA1 | 0.34 | no | T[A/G]A | 143110762 | 143110501-143111500 | 2 | 8.89E-01 |
| rs9331896 | 8 | CLU | 0.38 | yes | C[A/G]A | 27467686 | 27467001-27468000 | 3 | 1.79E-02 |
| rs983392 | 11 | MS4A6A | 0.40 | yes | C[A/G]T | 59923508 | 59923001-59924000 | 2 | 2.66E-10 |
| rs10792832 | 11 | PICALM | 0.36 | no | G[A/G]A | 85867875 | 85867501-85868500 | 2 | 4.29E-01 |
| rs4147929 | 19 | ABCA7 | 0.19 | no | T[A/G]T | 1063443 | 1063001-1064000 | 2 | 5.47E-01 |
| rs3865444 | 19 | CD33 | 0.31 | no | T[G/T]G | 51727962 | 51727001-51728000 | 3 | 1.71E-01 |

**Supplementary Table 2**. Characteristic of the cohorts included in the study.

| Study | Consortium | cohort | case (N) | control (N) | total (N) |
| --- | --- | --- | --- | --- | --- |
| Discovery | ADGC | ACT | 524 | 1571 | 2095 |
|  |  | ADC1 | 1532 | 512 | 2044 |
|  |  | ADC2 | 623 | 156 | 779 |
|  |  | ADC3 | 731 | 568 | 1299 |
|  |  | ADC4 | 304 | 377 | 681 |
|  |  | ADC5 | 286 | 505 | 791 |
|  |  | ADC6 | 213 | 338 | 551 |
|  |  | ADC7 | 514 | 790 | 1304 |
|  |  | ADNI | 268 | 173 | 441 |
|  |  | GSK | 652 | 712 | 1364 |
|  |  | LOAD | 722 | 951 | 1673 |
|  |  | MAYO | 658 | 1046 | 1704 |
|  |  | MIRAGE | 225 | 349 | 574 |
|  |  | MTV | 253 | 188 | 441 |
|  |  | OHSU | 132 | 153 | 285 |
|  |  | PFIZER | 696 | 762 | 1458 |
|  |  | ROSMAP | 295 | 769 | 1064 |
|  |  | TARC1 | 323 | 181 | 504 |
|  |  | TGEN2 | 617 | 365 | 982 |
|  |  | MIAMI | 1134 | 1119 | 2253 |
|  |  | UPITT | 1161 | 829 | 1990 |
|  |  | WASHU | 318 | 187 | 505 |
|  | Total |  | 12181 | 12601 | 24782 |
|  |  |  |  |  |  |
| Replication | CHARGE | AGES | 95 | 2708 | 2803 |
|  |  | ASPS | 277 | 169 | 446 |
|  |  | CHS | 450 | 1702 | 2152 |
|  |  | FHS | 330 | 3910 | 4240 |
|  |  | ROTT | 985 | 4985 | 5970 |
|  | EADI | EADI | 2240 | 6631 | 8871 |
|  | GERAD | GERAD | 3177 | 7277 | 10454 |
|  | Total |  | 7554 | 27382 | 34936 |
| Total |  |  | 19735 | 39983 | 59718 |

**Supplementary Table 3.** Distribution of age and sex in datasets for mQTL and eQTL analysis^1^

|  | ROSMAP  mQTL | ROSMAP  eQTL | FHS  mQTL | FHS  eQTL |
| --- | --- | --- | --- | --- |
| N | 740 | 580 | 2405 | 5252 |
| Female (%) | 471 (63.6%) | 367 (63.3%) | 1306 (54%) | 2833 (54%) |
| Age | 88 (7) | 89 (7) | 66 (9) | 55 (13) |

^1^Tissue types of datasets are brains in ROSMAP and blood in FHS.

Abbreviations: ROSMAP, Religious Order Study and the Memory and Aging Project; FHS, Framingham Heart Study; mQTL, methylation quantitative trait locus; eQTL, expression quantitative trait locus.

| **Supplementary Table 4.** Comparisons between CGS and non-CGS in each top AD-associated window in the discovery stage | | | | | | | | | | | | | |
| --- | --- | --- | --- | --- | --- | --- | --- | --- | --- | --- | --- | --- | --- |
|  |  |  |  | Window | | |  | SNP | | | | | |
| Chr | Start | End | Gene | Type | P^1^ | P^2^ |  | Type | rsID | Effect allele | MAF | OR (95% CI) | P |
| 2 | 127847001 | 127848000 | BIN1 | CGS | 1.27E-13 | 1.27E-13 |  | CGS | rs35114168 | A | 0.39 | 1.16 (1.12, 1.21) | 1.48E-13 |
|  |  |  |  |  |  |  |  | CGS | rs6718147 | A | 0.38 | 0.91 (0.88, 0.95) | 5.88E-06 |
|  |  |  |  | nonCGS | 3.30E-05 | 3.02E-05 |  | nonCGS | rs35589443 | C | 0.02 | 1.35 (1.17, 1.56) | 3.02E-05 |
|  |  |  |  |  |  |  |  | nonCGS | rs192628066 | T | 0.004 | 1.47 (0.82, 2.63) | 1.97E-01 |
| 11 | 59923001 | 59924000 | MS4A6A | CGS | 2.66E-10 | 2.66E-10 |  | CGS | rs983392 | G | 0.39 | 0.88 (0.85, 0.91) | 1.41E-10 |
|  |  |  |  |  |  |  |  | CGS | rs12226022 | C | 0.4 | 0.88 (0.85, 0.92) | 1.07E-09 |
|  |  |  |  | nonCGS | 7.86E-01 | 7.86E-01 |  | nonCGS | rs10897009 | A | 0.4 | 0.88 (0.85, 0.92) | 5.16E-10 |
|  |  |  |  |  |  |  |  | nonCGS | rs652354 | T | 0.43 | 0.90 (0.87, 0.94) | 9.53E-08 |
|  |  |  |  |  |  |  |  | nonCGS | rs149882384 | C | 0.01 | 0.86 (0.65, 1.14) | 2.96E-01 |
|  |  |  |  |  |  |  |  | nonCGS | rs78763495 | A | 0.005 | 0.97 (0.73, 1.29) | 8.26E-01 |
| 11 | 60087501 | 60088500 | MS4A4A | CGS | 6.36E-10 | 6.36E-10 |  | CGS | rs4354705 | C | 0.36 | 0.87 (0.83, 0.90) | 8.44E-12 |
|  |  |  |  |  |  |  |  | CGS | rs4475941 | T | 0.34 | 1.09 (1.05, 1.14) | 9.71E-06 |
|  |  |  |  | nonCGS | 2.89E-02 | 1.07E-02 |  | nonCGS | rs4475940 | T | 0.34 | 1.09 (1.03, 1.16) | 2.59E-03 |
|  |  |  |  |  |  |  |  | nonCGS | rs4258399 | A | 0.34 | 1.09 (1.03, 1.16) | 2.59E-03 |
|  |  |  |  |  |  |  |  | nonCGS | rs4258400 | C | 0.34 | 1.09 (1.03, 1.16) | 2.59E-03 |
|  |  |  |  |  |  |  |  | nonCGS | rs4436562 | G | 0.34 | 1.09 (1.03, 1.16) | 2.59E-03 |
|  |  |  |  |  |  |  |  | nonCGS | rs11560910 | C | 0.34 | 1.09 (1.03, 1.16) | 2.59E-03 |
|  |  |  |  |  |  |  |  | nonCGS | rs10897034 | T | 0.29 | 1.06 (1.02, 1.11) | 4.89E-03 |
|  |  |  |  |  |  |  |  | nonCGS | rs182308188 | A | 0.01 | 0.79 (0.64, 0.98) | 3.09E-02 |
|  |  |  |  |  |  |  |  | nonCGS | rs11230249 | A | 0.03 | 1.16 (0.99, 1.35) | 7.11E-02 |
|  |  |  |  |  |  |  |  | nonCGS | rs2920389 | G | 0.0003 | 1.41 (0.29, 6.95) | 6.74E-01 |
| 11 | 85759501 | 85760500 | PICALM | CGS | 6.34E-09 | 6.34E-09 |  | CGS | rs694011 | T | 0.32 | 0.90 (0.86, 0.95) | 3.77E-05 |
|  |  |  |  |  |  |  |  | CGS | rs647422 | C | 0.43 | 1.03 (0.99, 1.07) | 1.13E-01 |
|  |  |  |  | nonCGS | 3.50E-02 | 4.20E-02 |  | nonCGS | rs111552865 | T | 0.24 | 1.09 (1.04, 1.14) | 1.65E-04 |
|  |  |  |  |  |  |  |  | nonCGS | rs112698952 | A | 0.24 | 1.09 (1.04, 1.14) | 1.71E-04 |
|  |  |  |  |  |  |  |  | nonCGS | rs111955441 | A | 0.24 | 1.09 (1.04, 1.14) | 1.81E-04 |
|  |  |  |  |  |  |  |  | nonCGS | rs12273113 | A | 0.04 | 0.88 (0.79, 0.98) | 1.67E-02 |
|  |  |  |  |  |  |  |  | nonCGS | rs149035717 | T | 0.001 | 1.46 (0.87, 2.46) | 1.48E-01 |
|  |  |  |  |  |  |  |  | nonCGS | rs187436656 | T | 0.002 | 0.74 (0.40, 1.35) | 3.26E-01 |
| 19 | 45411501 | 45412500 | APOE | CGS | 2.99E-46 | 2.99E-46 |  | CGS | rs429358 | C | 0.25 | 3.73 (3.53, 3.94) | <2.23e-308 |
|  |  |  |  |  |  |  |  | CGS | rs7412 | T | 0.07 | 0.56 (0.51, 0.62) | 3.56E-28 |
| ^1^P for window when all CGSes or all nonCGSes are included. ^2^P for window when those SNPs with minor allele frequency < 0.5% are excluded. | | | | | | | | | | | | | |

Note: For the top AD-associated windows, the P values of the windows under two conditions were shown, which are the analysis including only the CGSes and only nonCGSes. The detailed summary statistics of each SNP in each window were also presented.

Abbreviations: SNP, single nucleotide polymorphism; CGS, CpG-related SNP; nonCGS, SNPs not related to CpG dinucleotides; MAF, minor allele frequency.

| **Supplementary Table 5.** Genes showing nominal association with DNA methylation level of cg14750746 in brain (P<0.005) | | | | | |  |
| --- | --- | --- | --- | --- | --- | --- |
| Chr | Start | End | Gene ID | Gene Name | P | |
| chr1 | 16542404 | 16543823 | ENSG00000237276.3 | *ANO7L1* | 4.34E-03 | |
| chr2 | 85825671 | 85830319 | ENSG00000168890.9 | *TMEM150A* | 2.79E-03 | |
| chr2 | 88469835 | 88486146 | ENSG00000144115.12 | *THNSL2* | 2.52E-03 | |
| chr2 | 124782864 | 125672864 | ENSG00000155052.14 | *CNTNAP5* | 4.93E-03 | |
| chr2 | 175199674 | 175203220 | ENSG00000217236.1 | *SP9* | 4.67E-03 | |
| chr3 | 50606583 | 50622366 | ENSG00000114735.5 | *HEMK1* | 4.65E-03 | |
| chr4 | 53588785 | 53617807 | ENSG00000226887.3 | *ERVMER34-1* | 4.33E-03 | |
| chr5 | 156564423 | 156586030 | ENSG00000155868.7 | *MED7* | 3.10E-03 | |
| chr6 | 31629006 | 31634060 | ENSG00000204438.5 | *GPANK1* | 4.38E-03 | |
| chr7 | 102122892 | 102184211 | ENSG00000170667.9 | *RASA4B* | 1.54E-03 | |
| chr7 | 102220093 | 102283316 | ENSG00000105808.11 | *RASA4* | 3.03E-03 | |
| chr7 | 102277472 | 102283238 | ENSG00000267368.1 | *UPK3BL* | 9.16E-04 | |
| chr8 | 67955314 | 67996018 | ENSG00000121022.9 | *COPS5* | 3.23E-03 | |
| chr8 | 94710789 | 94743755 | ENSG00000188343.8 | *FAM92A1* | 2.22E-03 | |
| chr8 | 107771711 | 107782473 | ENSG00000174429.3 | *ABRA* | 3.54E-03 | |
| chr9 | 99518147 | 99540411 | ENSG00000081386.8 | *ZNF510* | 4.62E-03 | |
| chr9 | 102741461 | 102861322 | ENSG00000023318.7 | *ERP44* | 2.76E-03 | |
| chr9 | 135906076 | 135933888 | ENSG00000148308.12 | *GTF3C5* | 1.73E-03 | |
| chr10 | 52499078 | 52576255 | ENSG00000204147.5 | *ASAH2B* | 1.60E-03 | |
| chr11 | 85563600 | 85565986 | ENSG00000215504.2 | *AP000974.1* | 4.64E-03 | |
| chr12 | 95910336 | 95945266 | ENSG00000136014.7 | *USP44* | 2.82E-03 | |
| chr13 | 99102455 | 99230194 | ENSG00000102572.10 | *STK24* | 4.48E-03 | |
| chr13 | 111968531 | 111996596 | ENSG00000153495.6 | *TEX29* | 1.75E-03 | |
| chr14 | 57735627 | 57756797 | ENSG00000053770.7 | *AP5M1* | 2.02E-03 | |
| chr15 | 57884139 | 57977562 | ENSG00000263155.1 | *GCOM1* | 4.59E-03 | |
| chr16 | 69165194 | 69202941 | ENSG00000141076.12 | *CIRH1A* | 3.00E-03 | |
| chr17 | 66031635 | 66042958 | ENSG00000182481.4 | *KPNA2* | 2.50E-03 | |
| chr19 | 4324041 | 4338847 | ENSG00000178078.5 | *STAP2* | 2.89E-03 | |
| chr19 | 4502204 | 4517716 | ENSG00000167676.3 | *PLIN4* | 2.14E-03 | |
| chr19 | 44010872 | 44031396 | ENSG00000105755.2 | *ETHE1* | 6.44E-04 | |
| chr19 | 48867657 | 48879627 | ENSG00000105467.3 | *SYNGR4* | 4.40E-03 | |
| chr19 | 55851221 | 55859488 | ENSG00000133247.9 | *SUV420H2* | 1.99E-03 | |
| chr19 | 55851254 | 55856561 | ENSG00000267531.1 | *AC020922.1* | 5.49E-04 | |
| chr22 | 23950639 | 23974508 | ENSG00000189269.7 | *C22orf43* | 1.08E-03 | |

| **Supplementary Table 6.** Pearson correlation between reported AD associated methylation site by De Jager et al., 2014[4] and the site reported in the current study in both brains and blood | | | | | | | | | | | |
| --- | --- | --- | --- | --- | --- | --- | --- | --- | --- | --- | --- |
| Chr | Reported CpG site associated with AD | |  | CpG site in the current study | |  | Brain | |  | Blood | |
|  | pos | name |  | pos | name |  | r | *P* |  | r | *P* |
| 2 | 127800646 | cg22883290 |  | 127862614 | cg00436254 |  | 0.04 | 0.263 |  | 0.04 | 0.04 |
| 11 | 72532891 | cg21806242 |  | 59824541 | cg01917716 |  | 0.08 | 0.03 |  | -0.07 | 1.33E-04 |
| 11 | 72532891 | cg21806242 |  | 60101475 | cg14750746 |  | 0.21 | 1.65E-08 |  | -0.08 | 2.13E-05 |
| 11 | 72532891 | cg21806242 |  | 85566560 | cg15822411 |  | 0.32 | < 2.2E-16 |  | 0.05 | 0.01 |
| 19 | 44278628 | cg22904711 |  | 45395297 | cg02613937 |  | -0.09 | 0.01 |  | 0.11 | 4.01E-09 |
| Note: Sample size are 2648 and 740 for Framingham blood and ROSMAP brain methylation data, which include all the methylation data without filtering by missing data of SNP or gene expression. | | | | | | | | | | | |

| **Supplementary Table 7.** Conditional analysis of the dosage effect of CGS on AD risk adjusted for the top GWAS SNP in the region | | | | | | | | | | | | | | |  |  |
| --- | --- | --- | --- | --- | --- | --- | --- | --- | --- | --- | --- | --- | --- | --- | --- | --- |
| Top windows | | | | | |  | Reported top GWAS SNP | | | | |  | P value comparisons | | | |
| Chr | Start | End | Region or Closest Gene | N of CGS | top CGS in the window |  | rsID | CGS annotation | Reference | Distance to top CGS in the window (kb) | LD with top CGS in the window (R^2^) |  | Window^1^ | Window^2^ | GWAS SNP^1^ | GWAS SNP^2^ |
| 2 | 127847001 | 127848000 | *BIN1* | 2 | rs35114168 |  | rs6733839 | no | Lambert et al., 2013 | 45 | 0.27 |  | 2.14E-03 | 0.35 | 7.41E-19 | 5.51E-17 |
|  |  |  |  |  |  |  | rs7561528 | yes | Naj et al., 2011 | 42 | 0.89 |  | 2.14E-03 | 0.7941 | 8.92E-14 | 5.90E-12 |
| 11 | 59923001 | 59924000 | *MS4A6A* | 2 | rs983392 |  | rs983392 | yes | Lambert et al., 2013 | 0 | NA |  | 3.40E-10 | NA | NA | NA |
| 11 | 60087501 | 60088500 | *MS4A4A* | 2 | rs4354705 |  | rs4938933 | no | Naj et al., 2011 | -54 | 0.56 |  | 2.67E-10 | 5.59E-03 | 7.48E-11 | 1.54E-03 |
| 11 | 85759501 | 85760500 | *PICALM* | 2 | rs694011 |  | rs10792832 | no | Lambert et al., 2013 | 108 | 0.62 |  | 9.28E-05 | 0.9165 | 2.15E-10 | 6.79E-07 |
|  |  |  |  |  |  |  | rs561655 | yes | Naj et al., 2011 | 40 | 0.83 |  | 9.28E-05 | 0.2481 | 8.77E-10 | 1.23E-06 |
| 19 | 45411501 | 45412500 | *APOE* | 2 | rs429358 |  | rs429358 | yes | Lambert et al., 2013 | 0 | NA |  | 2.77E-556 | NA | NA | NA |
| ^1^The *P*-values of the window and reported top GWAS SNP are obtained from two regression models including either the dosage of CGSes in the window or reported top GWAS SNP with the adjustment of age, sex, and PCs across 24 European ADGC cohorts included in the discovery stage.  ^2^The *P*-values of the window and reported top GWAS SNP are obtained from only one regression model including both the dosage of CGSes in the window and reported top GWAS SNP with the adjustment of age, sex, and PCs across 24 European ADGC cohorts included in the discovery stage. | | | | | | | | | | | | | | | | |

**Figures:**

**Supplementary Figure 1**. Quantile-Quantile (Q-Q) plot of sliding window results in the discovery stage:


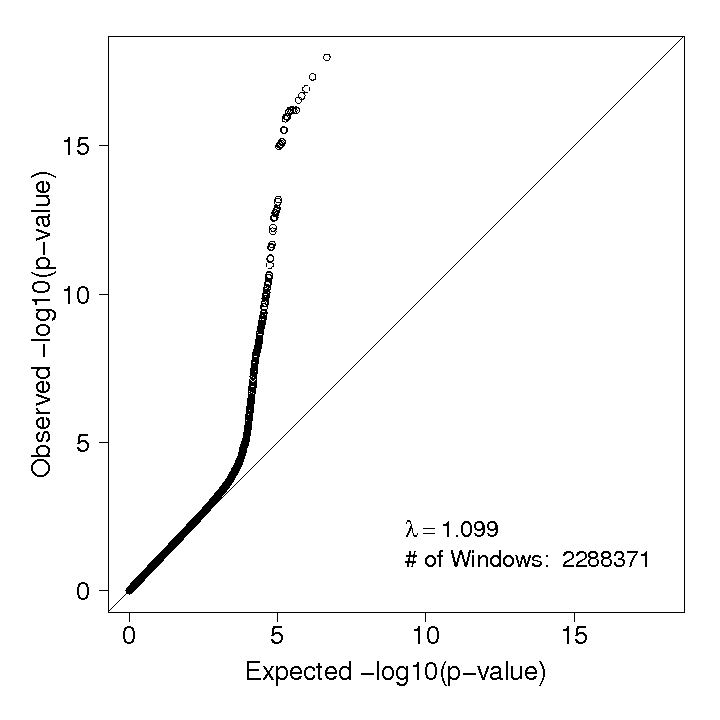


**Supplementary Figure 2**. Miami plot of the P values for the associations between each window on AD risk obtained from SKAT-O (upper panel) and CG dosage tests (lower panel) in discovery stage.


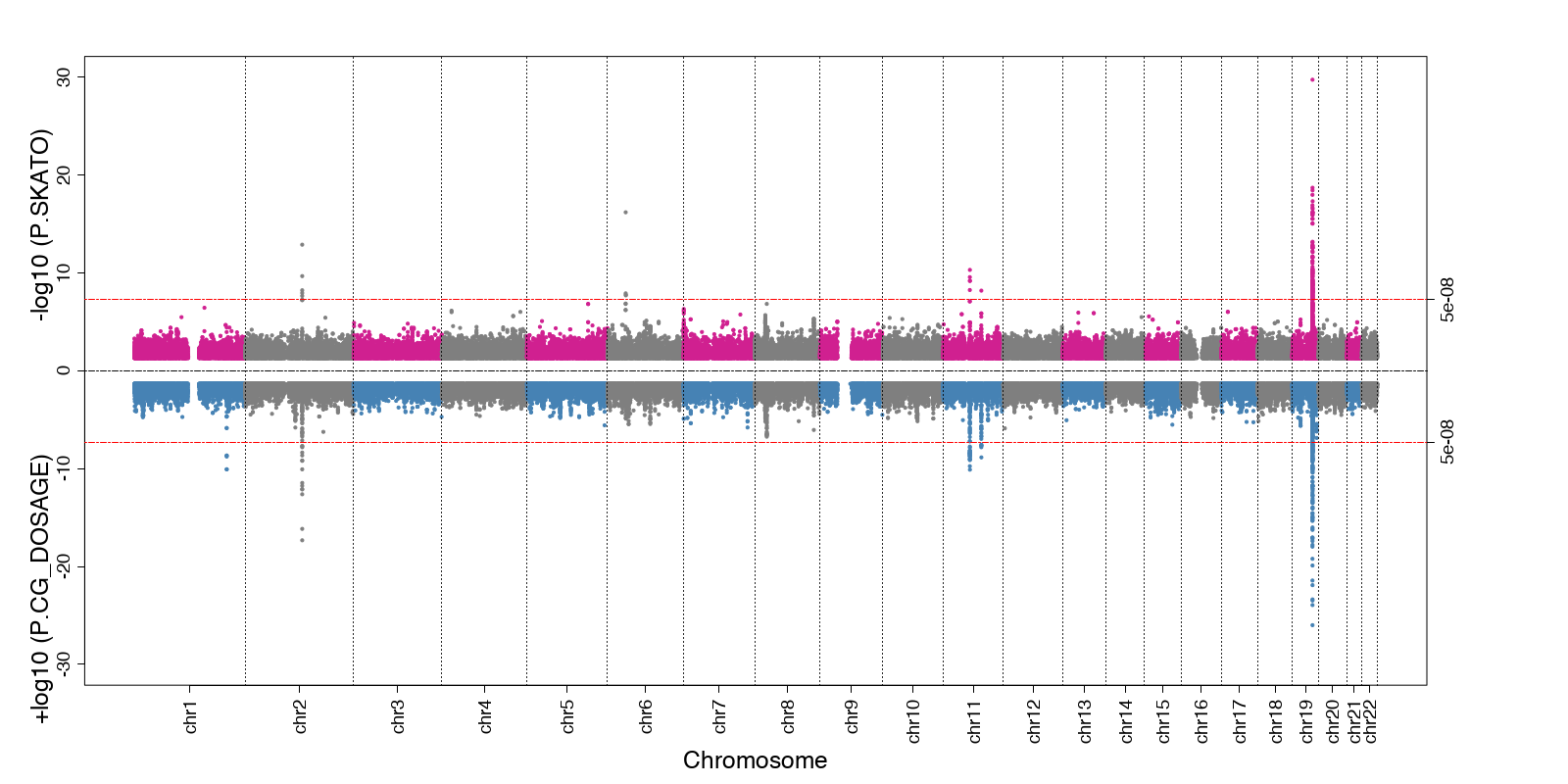


**BIN1**

**Intergenic,**

**LRFN2 (270k) – UNC5CL (175k)**

**TREM2**

**Intergenic,**

**LRFN2 (270k) – UNC5CL (175k)**

**Intergenic regions,**

**MS4A4E (30k) – MS4A4A (17k)**

**MS4A2 (57k) – MS4A6A (17k)**

**MS4A4A (10k) – MS4A6E (16k)**

**PICALM**

**APOE**

**CR1**

**Supplementary Figure 3.** Regional plots of top AD-associated windows in discovery stage: (A) *BIN1*; (B) *MS4A* cluster; (C) *PICALM*. Each dot represents one window, and the top SNP within the top window across the whole region was labeled. The color represents the LD between widows, which was calculated between the top SNPs within each window.

C

B


A

**Reference:**

1. Beekly DL, Ramos EM, Lee WW, Deitrich WD, Jacka ME, Wu J, et al. The National Alzheimer's Coordinating Center (NACC) database: the Uniform Data Set. Alzheimer Dis Assoc Disord. 2007;21(3):249-58. doi: 10.1097/WAD.0b013e318142774e. PubMed PMID: 17804958.

2. Morris JC, Weintraub S, Chui HC, Cummings J, Decarli C, Ferris S, et al. The Uniform Data Set (UDS): clinical and cognitive variables and descriptive data from Alzheimer Disease Centers. Alzheimer Dis Assoc Disord. 2006;20(4):210-6. doi: 10.1097/01.wad.0000213865.09806.92. PubMed PMID: 17132964.

3. Lambert JC, Ibrahim-Verbaas CA, Harold D, Naj AC, Sims R, Bellenguez C, et al. Meta-analysis of 74,046 individuals identifies 11 new susceptibility loci for Alzheimer's disease. Nat Genet. 2013;45(12):1452-8. doi: 10.1038/ng.2802. PubMed PMID: 24162737; PubMed Central PMCID: PMCPMC3896259.

4. De Jager PL, Srivastava G, Lunnon K, Burgess J, Schalkwyk LC, Yu L, et al. Alzheimer's disease: early alterations in brain DNA methylation at ANK1, BIN1, RHBDF2 and other loci. Nat Neurosci. 2014;17(9):1156-63. doi: 10.1038/nn.3786. PubMed PMID: 25129075; PubMed Central PMCID: PMCPMC4292795.
